# Impact of atrial fibrillation diagnosis-to-ablation time on 24-month efficacy and safety outcomes in the Cryo Global Registry

**DOI:** 10.1093/europace/euaf008

**Published:** 2025-01-21

**Authors:** Dennis Lawin, Christoph Stellbrink, Kyoung-Ryul Julian Chun, Cheng-Hung Li, Kelly A van Bragt, Fred Kueffer, Jada M Selma, Il-Young Oh, Jean Manuel Herzet, Junichi Nitta, Ting Yung Chang, Thorsten Lawrenz

**Affiliations:** Department of Cardiology and Intensive Care Medicine, Bielefeld University, Medical School and University Medical Center OWL, Public Hospital of Bielefeld, Teutoburger Str. 50, D-33604 Bielefeld, Germany; Department of Cardiology and Intensive Care Medicine, Bielefeld University, Medical School and University Medical Center OWL, Public Hospital of Bielefeld, Teutoburger Str. 50, D-33604 Bielefeld, Germany; Cardioangiologisches Centrum Bethanien, Frankfurt, Germany; Taichung Veterans General Hospital, Taichung, Taiwan; Medtronic, Inc., Minneapolis, MN, USA; Medtronic, Inc., Minneapolis, MN, USA; Medtronic, Inc., Minneapolis, MN, USA; Department of Internal Medicine, Seoul National University Bundang Hospital, Seoul, Republic of Korea; Centre Hospitalier Régional de la Citadelle, Liège, Belgium; Sakakibara Heart Institute, Fuchu, Japan; Taipei Veterans General Hospital, Taipei City, Taiwan; Department of Cardiology and Intensive Care Medicine, Bielefeld University, Medical School and University Medical Center OWL, Public Hospital of Bielefeld, Teutoburger Str. 50, D-33604 Bielefeld, Germany

**Keywords:** Atrial fibrillation, Catheter ablation, Cryoballoon, Early ablation, Registry

## Abstract

**Aims:**

Early rhythm control therapy in atrial fibrillation (AF) results in higher freedom from atrial arrhythmia (AA) recurrence and improved cardiovascular outcomes. The optimal timing of cryoballoon ablation (CBA) is unknown.

**Methods and results:**

We evaluated AA recurrence and procedure-related complications of early vs. late CBA (≤12 vs. >12 months from diagnosis) in patients enrolled in the prospective Cryo Global Registry (121 centres in 37 countries, NCT02752737). A total of 3447 subjects were followed through 12 months and 1220 through 24 months. In summary, 1573 patients (46%) had early ablation at a median (IQR) of 0.3 (0.1–0.6) years from AF diagnosis (age 62 ± 12 years., 35.8% female, 71.4% paroxysmal), and 1874 (54%) had late ablation at a median of 3.4 (1.9–6.7) years after diagnosis (age 61 ± 11 years, 36.2% female, 75.0% paroxysmal). Early ablation patients were less hypertensive (53.5% vs. 57.9%, *P* = 0.01) and less symptomatic (1.5 ± 1.1 vs. 1.8 ± 1.1 symptoms/patient, *P* < 0.01) and had smaller left atrial diameters (41 ± 7 mm vs. 42 ± 7 mm, *P* < 0.01). Freedom from AA recurrence was 81.5% (95% CI: 78.7–83.9%) in the early vs. 71.7% (95% CI: 68.9–74.3%) in the late ablation group at 24 months (*P* < 0.01). The risk of cardioversion was 41% lower in the early ablation group [HRAdj: 0.59 (0.42–0.83), *P* < 0.01]. Serious procedure-related adverse events occurred in 2.4 and 3.5% of patients in the early and late ablation groups (*P* = 0.045), respectively.

**Conclusion:**

CBA within 12 months from AF diagnosis resulted in higher freedom from AA recurrence and is associated with fewer safety events in a real-world evaluation.

**Clinical trial registration:**

https://clinicaltrials.gov/ct2/show/NCT02752737

## Graphical Abstract

**Graphical Abstract:**
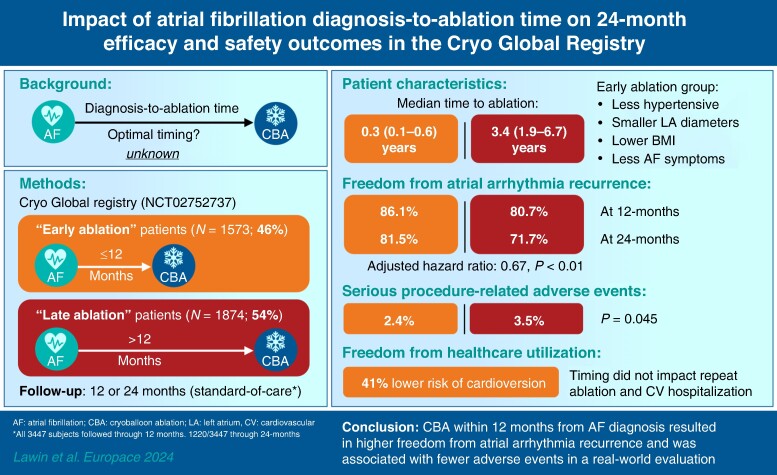

**What’s New?**

- Cryoballoon ablation of atrial fibrillation (AF) within 12 months from AF diagnosis resulted in higher freedom from atrial arrhythmia recurrence.
- Early ablation of AF was associated with fewer safety events in a real-world evaluation.

## Introduction

Atrial fibrillation (AF) is the most frequently diagnosed cardiac arrhythmia and is associated with high morbidity and mortality.^1,2^ The number of affected patients will substantially increase during the next years causing a major burden of health care costs.^3^ The strategy of rhythm control is still a concern of daily practice.^1^ Early treatment of AF seems reasonable because AF is causing progressive atrial fibrosis in the process of atrial remodelling related to AF duration.^4,5^ Progressive atrial remodelling contributes to lower success rates of rhythm therapy if initiated belatedly.^6^

Current evidence indicated that catheter ablation (CA) of AF resulted in higher freedom from atrial arrhythmia (AA) recurrence and better improvement of AF-related symptoms compared to antiarrhythmic drug (AAD) therapy in patients with paroxysmal AF (PAF).^7–10^ Additionally, first-line CA of PAF resulted in lower rates of progression to persistent AF (PsAF) compared to initial AAD therapy.^11,12^ Of note, the risk of thrombo-embolic events and mortality was increased in patients with PsAF compared to PAF in a sub-study of the ROCKET-AF trial which supports early rhythm control to stop AF progression.^13^ Nevertheless, some physicians and patients still postpone CA of AF due to fears regarding procedure-related adverse events but, of note, in a recently published observational cohort study, 19.2% of patients on a waitlist for AF ablation experienced significant morbidity allowing speculation that affected patients may experience disadvantages by delaying CA.^14^ However, earlier randomized clinical trials were not able to show prognostic improvement by rhythm control therapy over rate control so far.^15,16^ In contrast, the recently published EAST-AFNET 4 study indicated that especially early rhythm control therapy of AF was superior to usual care regarding the reduction of major cardiovascular endpoints and mortality.^17^ Of note, only a small proportion of patients in the EAST-AFNET 4 study were treated with CA (8%) for AF at the beginning of the trial while the majority of patients received AAD therapy. However, at the end of the 2-year period of the trial, the proportion of patients treated with CA increased to 19.4%.^17^

There is only little evidence indicating the reduction of a composite outcome of all-cause death, stroke, and major bleeding by early CA of AF compared to medical therapy alone.^18^ Still, the optimal timing of CA relative to AF diagnosis is unknown, and data on efficacy and safety outcomes of an early ablation strategy in a real-world setting over a longer period of time are lacking. Therefore, we investigated the efficacy and safety of early cryoballoon ablation (CBA) ≤ 12 months from first AF diagnosis compared to late CBA > 12 months from first AF diagnosis in a sub-study from the prospective, multicentre Cryo AF Global Registry.

## Methods

### Study design and cryoballoon ablation procedure

Patients were prospectively enrolled in the ongoing, multicentre, post-market Cryo Global Registry (NCT02752737) with the aim of evaluating global outcomes of CBA performed with the Arctic Front Advance Family of cryoablation catheters (Medtronic, Inc.) for the treatment of AF. Data collection followed the principles outlined in the Declaration of Helsinki (2013) and Good Clinical Practices. A local institutional review board or ethics committee at each participating centre approved the study, and all patients signed an informed consent form prior to participating. A global steering committee of international physicians oversees data quality, statistical analyses, and publication milestones.

Procedures within the Cryo Global Registry were performed according to the participating centre’s standard of care and have been described in detail before.^19,20^ Briefly, the 23 or 28 mm CBA catheter (Arctic Front Advance; Arctic Front Advance Pro; Medtronic, Inc.) was guided into the left atrium using a dedicated 15-F steerable sheath (FlexCath or FlexCath Advance Steerable Sheath; Medtronic, Inc.) and positioned at the antrum of the targeted pulmonary veins (PVs) using a J-tip wire or dedicated inner-lumen circular mapping catheter (Achieve or Achieve Advance, Medtronic, Inc.). It was protocol-prescribed to demonstrate PVI by entrance and/or exit block following the ablation and phrenic nerve monitoring was recommended during right-sided CBA. Acute success was defined as the ability to electrically isolate all targeted PVs. The number of cryoapplications per PV was determined by the operator. Adjunctive imaging, monitoring, and/or additional ablation targets (e.g. LA AF trigger, RA AF trigger, superior vena cava trigger, mitral valve isthmus, or line, left-sided roofline, complex fractionated atrial electrograms) using any commercially available catheter were used at the operator’s discretion. Patients’ discharge followed the hospital’s standard of care procedures.

### Patient population and follow-up

The inclusion criteria for the Cryo Global Registry were (i) patients aged 18 years or older and (ii) undergoing a planned CBA procedure. The exclusion criteria were patients (1) participating in a concurrent, unapproved trial or (ii) were unable to participate according to local laws. Additionally, the current analysis included a subset of patients that met the following criteria; patients with a minimum of 12 months of follow-up were included, and patients with (i) missing AF diagnosis date at baseline and (ii) patients with prior atrial flutter (AFL) or PVI ablation were excluded.

The early ablation group was defined as CBA ≤ 12 months from their first AF diagnosis. Patients in the late ablation group underwent ablation >12 months after their first AF diagnosis.

Patients were followed through 12 or 24 months via telephone visits and/or in-person visits, and annual visits were protocol-required for the duration of follow-up. Rhythm monitoring during follow-up followed standard of care and included, but was not limited to, electrocardiograms (ECGs), Holter monitor, trans-telephonic monitor, insertable cardiac monitor, pacemaker, and/or implantable cardioverter defibrillator.

### Endpoints

Patient demographics and procedural characteristics were collected, as well as patient-reported predefined symptoms of AF (dizziness, palpitations, rapid heartbeat, dyspneoa, fatigue, syncope) and quality of life (QoL) at baseline and annual follow-up visits. Quality of life was measured using the EQ-5D-3L index score (ranging from 0 to 1, with 1 being the highest score). Long-term safety was assessed as the rate of serious adverse events. This included all events that led to death or to a serious deterioration in health that resulted in either a life-threatening illness or injury, a permanent impairment in body structure or function, in-patient or prolonged hospitalization, or medical intervention to prevent life-threatening illness or injury. Long-term efficacy was defined as freedom from ≥30 s of documented AA recurrence [defined as AF/ AFL/atrial tachycardia (AT)] between a 90-day blanking period and through 24 months of follow-up. Recurrence of AF/AFL/AT at 12 months and 24 months was studied in additional subgroups: PAF and PsAF, left atrial diameter (LAD) < 50 mm and LAD ≥ 50 mm, and PVI and ablations beyond PVI (PVI+). Patients with AF episodes of AF <7 days in duration were classified with PAF, and patients with AF duration of ≥7 days and ≤ 12 months (PsAF) and patients with AF duration >12 months (longstanding PsAF (LSPsAF) were pooled into the PsAF cohort. Repeat ablations, cardioversions, and cardiovascular hospitalization rates were assessed through 12- and 24-month follow-ups. Improvement in AF symptoms and QoL was assessed at 12- and 24-month follow-ups and compared to baseline values.

### Statistics

The purpose of this analysis was to summarize ablation within 1 year of diagnosis and ablation above 1 year of diagnosis populations and outcomes. Continuous variables are summarized as mean ± standard deviation, and dichotomous variables are presented as numbers and per cent of subjects. Baseline and procedural characteristics are compared between ablation within 12 months of diagnosis and ablation above 12 months of diagnosis cohorts with a two-sample *t*-test for continuous variables and an exact test for dichotomous variables. For outcomes post-ablation, both unadjusted and adjusted analyses were completed. Adjusted models account for differences in baseline characteristics between the early and late ablation groups, utilizing propensity score methods. Kaplan–Meier methods were used to estimate freedom from ≥30 s AF/AFL/AT recurrence, repeat ablation, cardioversion, and cardiovascular-related rehospitalization. Standard error was calculated with Greenwood’s formula. Cox regression models were utilized to assess the hazard ratios for efficacy outcomes between cohorts. To account for differences in baseline characteristics between cohorts, propensity score methods were utilized to estimate an adjusted hazard ratio. Propensity scores were calculated for each subject with logistic regression, and propensity score was included as a covariate in the Cox regression model to estimate adjusted hazard ratios. Subjects missing baseline data had missing data imputed using multiple imputation methods. Multivariate imputation by fully conditional specification methods was utilized with the logistic regression method specified for classification variables and regression methods utilized for continuous variables. Similar methods were utilized for subgroup analysis. Cox regression with propensity score methods was utilized to assess efficacy in the subgroups PAF vs. PsAF, LAD ≤ 50 mm vs. LAD > 50 mm, and PVI only vs. PVI+. Propensity score, targeted subgroup, and interaction of early ablation indicator and targeted subgroup were included as covariates in the Cox regression model to estimate adjusted hazard ratios.

Changes in QoL and improvement in the number of AF-related symptoms through 24 months between cohorts were assessed with a repeated measures mixed model. The percentage of subjects with a serious procedure-related adverse event between cohorts was assessed with an exact test. PROC MI and PROC MIANALYZE were used to conduct multiple imputation analysis. Values of *P* < 0.050 were considered significant. Statistical analyses were completed using SAS software version 9.4 (SAS Institute, Cary, NC, USA).

## Results

### Patient characteristics

A total of 3447 patients were included in this analysis. Patients were enrolled at 121 centres in 37 countries and were followed for a minimum of 12 months. Of these patients, 1573 (46%) were ablated within 12 months from primary AF diagnosis (early ablation). A subset of 1220 patients from 39 centres in 20 countries was followed up for 24 months per the local standard of care, of which 464 (38%) were ablated within 12 months from diagnosis. Patient disposition is available in Supplementary material online, *Figure S1*.

Patients in the early ablation group had a median (IQR) of 0.3 (0.1–0.6) years from AF diagnosis, compared to a median of 3.4 (1.9–6.7) years after diagnosis (*P* < 0.01) in the late ablation group. Patients in the early ablation group had a significantly lower BMI (27 ± 5 vs. 28 ± 5; *P* < 0.01), smaller LA diameters (41 ± 7 vs. 42 ± 7 mm; *P* < 0.01), and a lower mean number of previously failed AAD (0.5 ± 0.6 vs. 0.9 ± 0.8; *P* < 0.01). A smaller proportion of patients in the early ablation group had a medical history of hypertension (53.5 vs. 57.9%; *P* = 0.01) and prior cardiac devices (3.1 vs. 4.9%; *P* < 0.01). Other baseline characteristics and comorbidities were not significantly different between both groups (*Table 1*).

**Table 1:** Patient baseline characteristics

	Ablation ≤ 12 months from diagnosis (*N* = 1573)	Ablation >12 months from diagnosis (*N* = 1874)	*P*-value^a^
Time from AF diagnosis to ablation (years)			
Mean ± SD	0.4 ± 0.3	5.3 ± 5.1	<0.01
Median (IQR)	0.3 (0.1–0.6)	3.4 (1.9–6.7)	
Male	1010 (64.2%)	1196 (63.8%)	0.83
Age	62 ± 12	61 ± 11	0.24
Body mass index (kg/m^b^)	27 ± 5	28 ± 5	<0.01
AF type			0.02
Paroxysmal AF	1123(71.4%)	1405 (75.0%)	
Persistent AF^b^	449 (28.5%)	469^b^ (25.0%)	
Left atrial diameter (mm)	41 ± 7	42 ± 7	<0.01
Left ventricular ejection fraction (%)	60 ± 9	60 ± 9	0.25
Number of previously failed antiarrhythmic drugs	0.5 ± 0.6	0.9 ± 0.8	<0.01
Number of first-line cryoballoon patients	697 (44.3%)	429 (22.9%)	
History of atrial flutter	98 (6.2%)	138 (7.4%)	0.20
History of atrial tachycardia	24 (1.5%)	34 (1.8%)	0.60
CHA_2_DS_2_-VASc	2.1 ± 1.6	2.0 ± 1.5	0.38
Baseline NYHA			0.24^c^
Class I	215 (13.7%)	250 (13.3%)	
Class II	185 (11.8%)	205 (10.9%)	
Class III	52 (3.3%)	54 (2.9%)	
Class IV	0 (0.0%)	0 (0.0%)	
Patient does not have heart failure (*N*, %)	999 (63.5%)	1225 (65.4%)	
NYHA status not reported (*N*, %)	121 (7.7%)	140 (7.5%)	
Hypertension	842 (53.5%)	1085 (57.9%)	0.01
Prior cardiac device	49 (3.1%)	92 (4.9%)	<0.01
Prior myocardial infarction (MI)	35 (2.2%)	61 (3.3%)	0.08
Prior stroke/transient ischaemic attack (TIA)	100 (6.4%)	110 (5.9%)	0.57
History of coronary artery disease	152 (9.7%)	216 (11.5%)	0.09
Diabetes	227 (14.4%)	255 (13.6%)	0.49
Sleep apneoa	72 (4.6%)	92 (4.9%)	0.69

AF, atrial fibrillation; IQR, interquartile range; NYHA, New York Heart Association.

^a^Two-sample *t*-test for continuous variables. Exact test for categorical variables.

^b^Persistent AF (*n* = 356) and longstanding persistent AF (*n* = 113) pooled.

^c^Wilcoxon rank-sum test. Numeric classifications were assigned as follows: no heart failure = 0, NYHA I = 1, NYHA II = 2, NYHA III = 3, NYHA IV = 4.

### Procedural characteristics

*Table 2* depicts the ablation procedure characteristics. Acute ablation success was high overall: 97.1% in the early ablation cohort and 95.2% in the late ablation cohort (*P* < 0.01). Total procedure time from venous access to the last cryocatheter removal (79 ± 34 vs. 83 ± 34 min; *P* < 0.01) and LA dwell time (53 ± 24 vs. 56 ± 25 min; *P* < 0.01) were shorter in the early ablation cohort, whereas total fluoroscopy time (35 ± 36 vs. 28 ± 28 min; *P* < 0.01) was longer. Conscious sedation was used in 58.0% of the early ablation cohort and 62.8% of the late ablation group (*P* < 0.01). The rates of pre-procedural mapping (CT or MRI), intracardiac echocardiography (ICE), and phrenic nerve monitoring were not different between cohorts. However, more esophageal temperature monitoring was observed in the early ablation cohort (49.8 vs. 38.6%; *P* < 0.01).

**Table 2:** Procedure characteristics

	Ablation ≤ 12 months from diagnosis (*N* = 1573)	Ablation >12 months from diagnosis (*N* = 1874)	*P*-value^a^
Total lab occupancy time (min)	139 ± 57	138 ± 51	0.53
Total procedure time (min, venous access to venous closure)	97 ± 42	97 ± 42	0.78
Total procedure time (min, venous access to last cryocatheter removal)	79 ± 34	83 ± 34	<0.01
Left atrial dwell time (min, first cryocatheter in to last cryocatheter removal)	53 ± 24	56 ± 25	<0.01
Total cryo fluoroscopy time (Min)	35 ± 36	28 ± 28	<0.01
Sedation method			<0.01
General	661 (42.0%)	696 (37.1%)	
Conscious	912 (58.0%)	1177 (62.8%)	
3D electroanatomical mapping	336 (21.4%)	263 (14.0%)	<0.01
Pre-procedural mapping CT or MRI	401 (25.5%)	425 (22.7%)	0.055
Intracardiac echocardiography	431 (27.4%)	518 (27.6%)	0.88
Phrenic nerve monitoring	1559 (99.1%)	1860 (99.3%)	0.71
Oesophageal temperature monitored (temperature probe)	784 (49.8%)	723 (38.6%)	<0.01
Cavotricuspid isthmus (CTI) ablation	248 (15.8%)	192 (10.2%)	<0.01
Non-PVI non-CTI ablation	166 (10.6%)	102 (5.4%)	<0.01
LA AF trigger	61 (3.9%)	67 (3.6%)	
RA AF trigger	21 (1.3%)	31 (1.7%)	
Superior vena cava trigger	17 (1.1%)	4 (0.2%)	
Mitral valve isthmus or line	5 (0.3%)	1 (0.1%)	
Left-sided roofline	67 (4.3%)	10 (0.5%)	
Complex fractionated atrial electrogram (CFAE)	2 (0.1%)	5 (0.3%)	
Rotor	0 (0.0%)	1 (0.1%)	
Other	34 (2.2%)	18 (1.0%)	
Focal RF PVI touch-up	29 (1.8%)	21 (1.1%)	0.09
Focal cryo PVI touch-up	3 (0.2%)	4 (0.2%)	1.0
Acute success	1522 (97.1%)	1781 (95.2%)	<0.01
Isoproterenol and/or adenosine use	279 (17.7%)	212 (11.3%)	<0.01

AF, atrial fibrillation; PVI, pulmonary vein isolation; RF, radiofrequency.

^a^Two-sample *t*-test for continuous variables. Exact test for categorical variables.

Early ablation patients had more ablations beyond PVI compared to the late ablation group; cavotricuspid isthmus (CTI) ablation was performed in 15.8% of patients in the early ablation cohort vs.10.2% in the late ablation group (*P* < 0.01), and additional non-PVI, non-CTI ablations were performed in 10.6% of the early ablation cohort vs. 5.4% in the late ablation group (*P* < 0.01). LA roofline was the most common PVI+ lesion in the early ablation cohort (4.3%), followed by LA AF trigger ablation (3.9%). In the late ablation group, LA AF trigger ablation (3.6%) was the most common adjunctive ablation target outside of PVI followed by RA AF trigger (1.7%) ablation.

### Long-term safety

A total of 113 serious procedure-related adverse events were reported in 103 patients. Early ablation was associated with a significantly lower rate of serious procedure-related adverse events (*P* = 0.045); 42 events were observed in 37 (2.4%) patients in the early ablation cohort, and 71 events were observed in 66 (3.5%) patients in the late ablation group. No atrio-esophageal fistula or pulmonary vein stenosis was reported; a full list of serious procedure-related adverse events can be found in *Table 3*.

**Table 3:** Listing of serious procedure adverse events

	Ablation ≤ 12 months of diagnosis	Ablation >12 months of diagnosis	*P*-value^a^
Total	42 (37, 2.4)	71 (66, 3.5)	0.045
Altered state of consciousness (due to sedation drug)	1 (1, 0.1)	0 (0, 0.0)	
Atrial septal defect	0 (0, 0.0)	1 (1, 0.1)	
Cardiac failure	1 (1, 0.1)	1 (1, 0.1)	
Cardiac tamponade, perforation, pericardial effusion^b^	5 (5, 0.3)	9 (9, 0.5)	
Embolic stroke	0 (0, 0.0)	1 (1, 0.1)	
Groin-site complication^c^	11 (10, 0.6)	20 (19, 1.1)	
Hypervolaemia	1 (1, 0.1)	0 (0, 0.0)	
Myocardial infarction or ischemic cardiac event^d^	2 (2, 0.1)	3 (3, 0.2)	
Other^e^	3 (3, 0.2)	2 (2, 0.1)	
Pericarditis	1 (1, 0.1)	2 (2, 0.1)	
Phrenic nerve injury	6 (6, 0.4)	10 (10, 0.5)	
Postoperative hypotension^f^	2 (2, 0.1)	2 (2, 0.1)	
Presyncope	0 (0, 0.0)	1 (1, 0.1)	
Pulmonary oedema	1 (1, 0.1)	0 (0, 0.0)	
Pulmonary or bronchial complication^g^	2 (2, 0.1)	6 (6, 0.3)	
Sepsis	0 (0, 0.0)	1 (1, 0.1)	
Stress cardiomyopathy	0 (0, 0.0)	1 (1, 0.1)	
Stroke or TIA of any cause^h^	1 (1, 0.1)	4 (4, 0.2)	
Supraventricular arrhythmias^i^	4 (4, 0.3)	7 (7, 0.4)	
Urinary retention	1 (1, 0.1)	0 (0, 0.0)	

TIA, transient ischaemic attack.

^a^Exact test.

^b^MedDRA terms: cardiac perforation (1), cardiac tamponade (8), pericardial effusion (5).

^c^MedDRA terms: arteriovenous fistula (6), arteriovenous fistula aneurysm (1), arteriovenous fistula site haematoma (1), femoral artery dissection (1), haematoma (2), incision site haematoma, puncture site discharge (1), puncture site haematoma (1), vascular access site haemorrhage (1), vascular pseudoaneurysm (12), vascular pseudoaneurysm ruptured (1), vessel puncture site discharge (1), vessel puncture site haematoma (1).

^d^MedDRA terms: angina pectoris (2), arteriospasm coronary (1), myocardial infarction (2).

^e^MedDRA terms: face injury (1), headache (1), lip injury (1), pyrexia (2).

^f^MedDRA terms: hypotension (2), post-procedural hypotension (2).

^g^MedDRA terms: haematemesis (2), haemoptysis (1), pleurisy (1), pneumonia (2), pneumothorax (1), pulmonary embolism (1).

^h^MedDRA terms: cerebral infarction (1), cerebrovascular accident (2), ischaemic stroke (1), lacunar stroke (1).

^i^MedDRA terms: atrial fibrillation (8), atrial tachycardia (1), nodal arrhythmia (1), sinus bradycardia (1).

### Long-term efficacy

**Figure 1:**
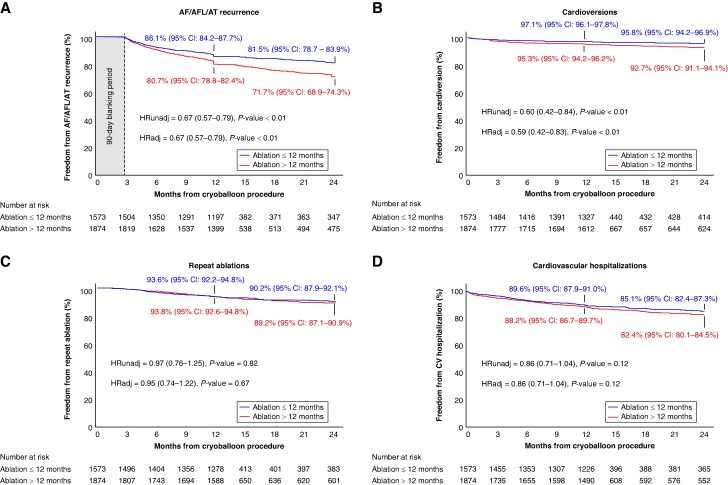
Effectiveness of cryoballoon ablation through 24 months follow-up. Kaplan–Meier graph of (*A*) rate of a ≥30 s recurrence of atrial fibrillation (AF)/atrial flutter (AFL)/atrial tachycardia (AT) after the blanking period through 24 months of follow-up, (*B*) cardioversions, (*C*) repeat ablations, and (*D*) cardiovascular hospitalizations throughout 24 months follow-up in patients treated with cryoballoon ablation (CBA) ≤ 12 months from AF diagnosis and >12 months from AF diagnosis. HRunadj represents the unadjusted hazard ratio, and HRadj represents the adjusted hazard ratio using propensity score-adjusted analysis for all baseline characteristics after multiple imputation of missing baseline data.

Patients undergoing CBA ≤ 12 months from primary diagnosis had a 33% lower risk of AF/AFL/AT recurrence at 24 months compared to patients with a diagnosis-to-ablation time >12 months, in a propensity score analysis adjusting for baseline characteristics [HRadj = 0.67 (0.57–0.79); *P* < 0.01, *Figure 1A*]. There were no statistical differences in the number of follow-up visits, ECGs, or Holters used between the early and late ablation groups (*Table 4*).

**Table 4:** Follow-up assessment within 24 months

	Ablation ≤ 12 months of diagnosis (*N* = 1573)	Ablation >12 months of diagnosis (*N* = 1874)	*P*-value^c^
Number of office visits completed through 12 months^a^	2.5 ± 1.8	2.4 ± 1.6	0.08
Number of ECGs reported 0–12 months	1.8 ± 1.9	1.7 ± 1.8	0.21
Number of Holter reported 0–12 months	0.8 ± 1.1	0.8 ± 1.1	0.26
Number of office visits completed 12–24 months^b^	1.3 ± 0.9	1.3 ± 0.9	0.21
Number of ECGs reported 12–24 months	0.8 ± 1.0	0.8 ± 1.0	0.98
Number of Holter reported 12–24 months	0.3 ± 0.5	0.3 ± 0.5	0.13

^a^Inclusive of 12-month required visit and any standard of care visit completed at the site post-ablation prior to 12-month study visit.

^b^Inclusive of 24-month required visit and any standard of care visit completed post 12-month study visit prior to 24-month visit.

^c^ *t*-test, testing for a difference between early vs. late CBA subgroups.

Subgroup analysis (*Table 5*) revealed that early ablation reduced the risk of AF/AFL/AT recurrence in all subgroups studied (PAF and PsAF, LAD <50 mm and LAD ≥50 mm, and PVI and PVI+). Overall, PsAF patients had higher recurrence than PAF patients at 12 and 24 months, but early ablation within 12 months post AF diagnosis reduced the risk of recurrence similarly in patients with both types of AF [HRadj = 0.69 (0.56–0.86) in PAF and HRadj = 0.65 (0.50–0.83) in PsAF, *P* = 0.67 between subgroups]. Similar observations were seen between patients with a LAD <50 mm [HRadj = 0.65 (0.54–0.79)] and LAD ≥ 50 mm [HRadj = 0.73 (0.45–1.16), *P* = 0.69 between subgroups].

**Table 5:** Subgroup analysis

Subgroup	Ablation timing	12-month efficacy	24-month efficacy	HRadj^a^ (95% CI)	*P*-value^b^
Paroxysmal AF	≤12 months	89.1% (95% CI: 87.0–90.8%)	85.0% (95% CI: 82.0–87.5%)	0.69 (0.56–0.86)	0.67
	>12 months	85.0% (95% CI: 83.0–86.8%)	77.2% (95% CI: 74.1–80.0%)		
Persistent AF	≤12 months	78.5% (95% CI: 74.2–82.2%)	71.0% (95% CI: 63.0–77.5%)	0.65 (0.50–0.83)	
	>12 months	68.1% (95% CI: 63.6–72.2%)	56.2% (95% CI: 50.3–61.8%)		
LAD < 50 mm	≤12 months	88.1% (95% CI: 85.9–90.1%)	84.3% (95% CI: 81.0–87.0%)	0.65 (0.54–0.79)	0.69
	>12 months	80.3% (95% CI: 77.8–82.6%)	73.4% (95% CI: 70.0–76.5%)		
LAD ≥ 50 mm	≤12 months	81.4% (95% CI: 71.7–88.0%)	69.4% (95% CI: 54.5–80.2%)	0.73 (0.45–1.16)	
	>12 months	70.6% (95% CI: 62.0–77.6%)	61.6% (95% CI: 50.7–70.7%)		
PVI only	≤12 months	83.7% (95% CI: 81.3–85.7%)	78.0% (95% CI: 74.6–81.1%)	0.77 (0.65–0.92)	<0.01
	>12 months	80.1% (95% CI: 78.0–82.0%)	70.6% (95% CI: 67.5–73.4%)		
PVI+	≤12 months	93.5% (95% CI: 90.4–95.6%)	91.8% (95% CI: 87.5–94.6%)	0.37 (0.23–0.60)	
	>12 months	83.9% (95% CI: 78.9–87.8%)	78.1% (95% CI: 71.3–83.5%)		

AF, atrial fibrillation; LAD, left atrial diameter; PVI, pulmonary vein isolation.

^a^Hazard ratio estimated by Cox regression, after adjusting for the difference in baseline characteristics between groups with propensity score methods. Subjects with missing baseline data had baseline data imputed with multiple imputation methods. Hazard ratios estimate the effect of ablation timing on AF/AFL/AT recurrence within each subgroup.

^b^Interaction term from Cox model. Indicates whether the effect of ablation timing on AF/AFL/AT recurrence is different between paroxysmal and persistent AF, between LAD < 50 mm and LAD ≥50 mm and between PVI only and PVI+.

In this real-world cohort, early ablation significantly reduced the risk of recurrence in both the PVI+ and PVI-only subgroups, but the treatment effect of early ablation was significantly higher in the PVI+ subgroup [HRadj = 0.37 (0.23–0.60)] compared to the PVI-only subgroup [HRadj = 0.77 (0.65–0.92), *P* < 0.01]. Overall, PVI+ patients had a higher freedom from AF/AFL/AT recurrence at 12 and 24 months compared to PVI-only patients. Treating patients with PVI+ within 12 months after diagnosis resulted in the highest freedom from AF/AFL/AT recurrence. Treating patients diagnosed with AF > 12 months with PVI+ resulted in similar success rates as treating patients early (≤12 months) with CBA-PVI alone.

### Repeat ablation, cardioversion, and hospitalization

Patients treated within 12 months after diagnosis had a 41% lower risk of undergoing cardioversion in the 24 months post-ablation [HRadj = 0.59 (0.42–0.83), *P* < 0.01] after a propensity-adjusted analysis (*Figure 1B*). The risk of repeat ablation [HRadj = 0.95 (0.74–1.22), *P* = 0.67, *Figure 1C*] and cardiovascular hospitalization [HRadj = 0.86 (0.71–1.04), *P* = 0.12, *Figure 1D*] were not statistically different between early and late ablation patients.

### Symptoms and quality of life

**Figure 2:**
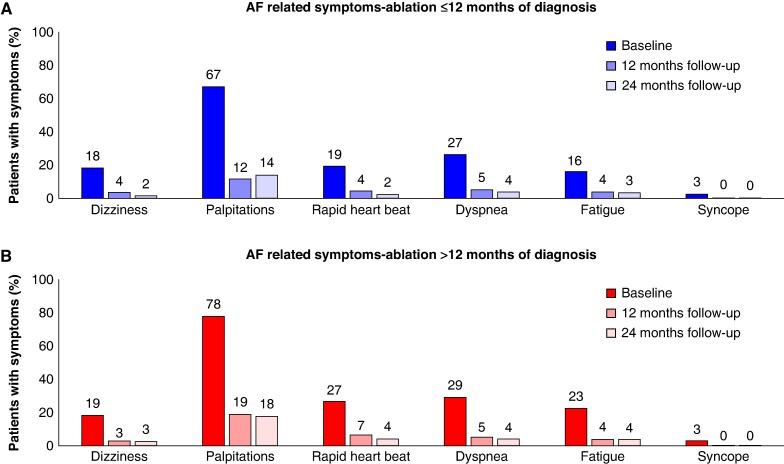
Atrial fibrillation (AF)-related symptom burden. Proportion of patients with predefined symptoms of AF at baseline, 12 months, and 24 months of follow-up receiving (*A*) cryoballoon ablation (CBA) ≤ 12 months from AF diagnosis and (*B*) CBA > 12 months from AF diagnosis.

At baseline, 84.0% of patients in the early ablation cohort experienced at least one AF-related symptom, with a mean number of 1.5 ± 1.1 symptoms per patient. In the cohort receiving ablation >12 months after diagnosis, 92.8% reported at least one symptom, with a mean of 1.8 ± 1.1 symptoms. Palpitation was the most reported symptom in both cohorts at baseline, followed by dyspnoea and rapid heartbeat (*Figure 2*). Symptoms were significantly reduced by CBA in both cohorts; at 24 months, 80.7% of the early ablation cohort and 76.7% of the late ablation group were free from predefined symptoms. After accounting for the difference in the mean number of symptoms at baseline, no clinically relevant difference was observed in the reduction of the number of symptoms between the early ablation and late ablation groups (reduction of 1.32 vs. 1.27 symptoms, respectively; *P* = 0.051).

The EQ-5D index score for overall QoL significantly improved from baseline to 12 and 24 months in both cohorts (*Table 6*), and there was no difference in the improvement between patients with a diagnosis-to-ablation time ≤12 months vs. >12 months.

**Table 6:** EQ-5D index score (0–1)

	Baseline		12-month follow-up		24-month follow-up			
	*N*	EQ-5D score	*N*	EQ-5D score	*N*	EQ-5D score	*P*-value^a^	*P*-value^b^
Ablation ≤12 months	1360	0.90 ± 0.14	1330	0.93 ± 0.12	424	0.94 ± 0.11	<0.01	0.38
Ablation >12 months	1686	0.88 ± 0.14	1663	0.93 ± 0.12	686	0.93 ± 0.12	<0.01	

All data available: subjects having baseline and at least one 12/24 month visit.

^a^Mixed model repeated measures, within cohort change over time.

^b^Mixed model repeated measures, comparing cohorts over time.

## Discussion

Our data shed important insights into efficacy and safety outcomes of early CBA ≤ 12 months from AF diagnosis in a large, global, real-world cohort of patients with PAF and PsAF. To summarize, early CBA was associated with a 33% lower risk of AA recurrence even in long-term follow-up. Not surprisingly, electrocardioversion was less often required in the early treatment group with a 41% lower risk compared to patients receiving CBA > 12 months from AF diagnosis. The improvement of arrhythmia-free survival by early CA of AF was previously shown by smaller studies and meta-analyses^21–23^ and is now confirmed referring to our large and global cohort of AF patients. Of note, the best evidence exists for early CA using thermal ablation techniques.^21–23^ The efficacy of novel techniques such as pulsed-field ablation for early CA of AF has yet to be investigated.^24^

Early CA of AF was associated with smaller LA diameters and better cardiovascular health at baseline indicating that early ablation stops the progression of atrial cardiomyopathy before atrial remodelling hampers ablations results. Of note, the lower risk of AA recurrence in patients undergoing CBA ≤ 12 months after primary AF diagnosis was confirmed by a propensity score analysis adjusting for baseline characteristics. Thus, early CBA of AF alone predicts better rhythm outcomes.

Interestingly, patients in the early CBA group have been applied to CBA only after a median of 0.3 years since AF diagnosis. Those patients were healthier than patients in the late ablation group indicated by a lower prevalence of hypertension, smaller LAD, fewer prior cardiac devices, and lower BMI. Thus, patients in the early CBA group may have been treated in centres having a lower threshold for CBA indication. This is supported by the facts that, first, patients in the early CBA group were asymptomatic in 16% of the cases; second, acute procedural success was higher in patients treated ≤12 months from AF diagnosis; and third, patients in the early CBA group more often received ablations beyond PVI including CTI ablation. Of note, subgroup analysis revealed that early ablation reduced the risk of AF/AFL/AT recurrence regardless of the type of AF (PAF and PSAF), LAD (< and ≥50 mm), or ablation approach (PVI and PVI+).

Nevertheless, the treatment effect of early ablation was significantly higher in patients receiving ablations beyond PVI with patients in the PVI+ ablation group having a higher freedom from AF/AFL/AT recurrence at 12 and 24 months compared to PVI-only patients. Treating patients diagnosed with AF > 12 months with PVI+ resulted in similar success rates as treating patients early (≤12 months) with PVI only reflecting that the higher burden of LA substrate in patients with longer diagnosis-to-ablation time can effectively be addressed by additional AF ablations. Patients with a diagnosis-to-ablation time ≤12 months after primary diagnosis, who were treated with PVI and additional ablations, had the highest freedom from AF/AFL/AT recurrence. However, the benefit of ablations beyond PVI is still an unsolved question with only a few studies indicating improved outcomes of patients treated with PVI and additional lesions.^25^ Therefore, the current guidelines are reluctant to recommend additional lesions.^1,26^ While empiric PVI+ approaches have not been shown to be superior to PVI only, personalized PVI+ strategies may be more effective, but this has to be investigated.

Importantly, the rate of serious adverse events was overall low in this large cohort of AF patients treated with CBA worldwide. On top of that, our analysis revealed that early CBA within 12 months after primary diagnosis was associated with significantly lower adverse events compared to late CBA. Thus, CBA should not unnecessarily be postponed due to safety reasons.

Up to now, randomized clinical trials have not been able to show prognostic improvement by rhythm control of AF over rate control so far.^15,16^ However, the EAST-AFNET 4 trial showed that early rhythm strategy was superior to usual care improving cardiovascular events and mortality.^17^ This is supported by other non-randomized trials revealing improved cardiovascular outcomes by early rhythm control within 12 months after primary AF diagnosis.^27^ Of note, nationwide real-world data from Taiwan indicated a benefit of a very early rhythm control strategy with a significant reduction of stroke, heart failure, acute myocardial infarction (MI), and mortality in patients treated within the first 3 months after primary diagnosis with AF with either CA or AAD therapy.^28^ Besides, there are other non-randomized data indicating an improvement of hard cardiovascular endpoints by early ablation of AF compared to medical therapy alone.^18^

The positive effects of early rhythm control within 12 months after primary diagnosis were also shown for patients with high comorbidity burden as assessed from analysis from large databases in that early CA or AAD therapy was shown to reduce a primary composite outcome of all-cause mortality, stroke, or hospitalization particularly in patients with CHA_2_DS_2_-VASc scores of 4 or higher.^29^ The same effect was shown by a sub-analysis from the EAST-AFNET 4 trial, in that patients with high comorbidity burden experienced a reduction of cardiovascular outcomes by early rhythm control within the first year after primary diagnosis.^30^ Moreover, recent data indicated that rhythm control with CA was shown to significantly reduce mortality and improve long-term outcomes in patients with heart failure.^31–33^

Referring to data from the Korean National Health Insurance Service database, early rhythm control of AF was associated with a lower risk of a composite outcome of death from cardiovascular causes, ischaemic stroke, admission to hospital for heart failure, or acute MI compared to rate control, but, as a matter of fact, this effect was not shown in patients who had been diagnosed with AF for more than 1 year before rhythm control.^34^ On the contrary, a sub-analysis of the AFFIRM trial was not able to show any superiority of early rhythm control within the first 6 months after primary AF diagnosis compared to rate control regarding several cardiovascular endpoints (survival, cardiovascular hospitalization, or ischaemic stroke).^35^ This raises the question whether the improvement of morbidity and mortality in newer trials may be rather due to refinement of AF management than to early timing of rhythm control.^35^

In spite of all data indicating improvement of AA-free survival and cardiovascular outcomes by early rhythm control, AF diagnosis-to-ablation time is highly dependent on patient care pathways, as many patients have AF well before an established chart recording. This is due to gaps in AF diagnosis. Better awareness for AF among patients and physicians as well as the use of health apps and smartwatches for rhythm detection will improve AF diagnosis.^36^ With a suspected increasing number of AF patients and higher awareness regarding CA of AF, wait time for ablation will increase continuously during the next years.^3,14^ Nevertheless, rhythm control should be applied early in all patients to delay AF progression as shown by the studies mentioned above. Thus, structures for AF ablation and treatment pathways should be improved to avoid long wait times, because longer wait times for CA of AF were shown to be associated with significant morbidity and adverse events.^14^

### Limitations

This analysis has some limitations. First, real-world registries usually go along with a selection bias. Patients in the early treatment group may have better cardiovascular health and less-progressive AF resulting in higher AA-free survival. Thus, we performed a propensity score analysis adjusting for baseline characteristics. Second, diagnosis-to-ablation time is heavily dependent on chart diagnosis of AF, and some patients likely have had AF before documented charting of AF in clinical history (especially those without symptoms). Third, rhythm monitoring was based on standard of care and not consistently performed with the same intensity among all centres. Thus, some AA relapses were underestimated.

## Conclusion

Our analysis emphasizes that CBA within 12 months from primary AF diagnosis results in higher freedom from AA recurrence and is associated with fewer safety events in a large, global real-world cohort of patients with PAF and PsAF. These results should encourage physicians to discuss the value of early CA of AF rather than the delay of care.

## Supplementary Material

euaf008_Supplementary_Data

## Data Availability

The data underlying this article are available in the article and in its online Supplementary material.

## References

[1] Hindricks G, Potpara T, Dagres N, Arbelo E, Bax JJ, Blomström-Lundqvist C et al 2020 ESC guidelines for the diagnosis and management of atrial fibrillation developed in collaboration with the European Association for Cardio-Thoracic Surgery (EACTS): the task force for the diagnosis and management of atrial fibrillation of the European Society of Cardiology (ESC) developed with the special contribution of the European Heart Rhythm Association (EHRA) of the ESC. Eur Heart J 2021;42:373–498. 32860505 10.1093/eurheartj/ehaa612

[2] Benjamin EJ, Wolf PA, D’Agostino RB, Silbershatz H, Kannel WB, Levy D. Impact of atrial fibrillation on the risk of death: the Framingham Heart Study. Circulation 1998;98:946–52. 9737513 10.1161/01.cir.98.10.946

[3] Krijthe BP, Kunst A, Benjamin EJ, Lip GY, Franco OH, Hofman A et al Projections on the number of individuals with atrial fibrillation in the European Union, from 2000 to 2060. Eur Heart J 2013;34:2746–51. 23900699 10.1093/eurheartj/eht280 PMC3858024

[4] Avitall B, Bi J, Mykytsey A, Chicos A. Atrial and ventricular fibrosis induced by atrial fibrillation: evidence to support early rhythm control. Heart Rhythm 2008;5:839–45. 18534368 10.1016/j.hrthm.2008.02.042

[5] Wijffels MC, Kirchhof CJ, Dorland R, Allessie MA. Atrial fibrillation begets atrial fibrillation. A study in awake chronically instrumented goats. Circulation 1995;92:1954–68. 7671380 10.1161/01.cir.92.7.1954

[6] Cosio FG, Aliot E, Botto GL, Heidbüchel H, Geller CJ, Kirchhof P et al Delayed rhythm control of atrial fibrillation may be a cause of failure to prevent recurrences: reasons for change to active antiarrhythmic treatment at the time of the first detected episode. Europace 2008;10:21–7. 18086696 10.1093/europace/eum276

[7] Wazni OM, Dandamudi G, Sood N, Hoyt R, Tyler J, Durrani S et al Cryoballoon ablation as initial therapy for atrial fibrillation. N Engl J Med 2021;384:316–24. 33197158 10.1056/NEJMoa2029554

[8] Andrade JG, Wells GA, Deyell MW, Bennett M, Essebag V, Champagne J et al Cryoablation or drug therapy for initial treatment of atrial fibrillation. N Engl J Med 2020;384:305–15. 33197159 10.1056/NEJMoa2029980

[9] Kuniss M, Pavlovic N, Velagic V, Hermida JS, Healey S, Arena G et al Cryoballoon ablation vs. antiarrhythmic drugs: first-line therapy for patients with paroxysmal atrial fibrillation. Europace 2021;23:1033–41. 33728429 10.1093/europace/euab029 PMC8286851

[10] Kanagaratnam P, McCready J, Tayebjee M, Shepherd E, Sasikaran T, Todd D et al Ablation versus anti-arrhythmic therapy for reducing all hospital episodes from recurrent atrial fibrillation: a prospective, randomized, multi-centre, open label trial. Europace 2023;25:863–72. 36576323 10.1093/europace/euac253 PMC10062288

[11] Andrade JG, Deyell MW, Macle L, Wells GA, Bennett M, Essebag V et al Progression of atrial fibrillation after cryoablation or drug therapy. N Engl J Med 2023;388:105–16. 36342178 10.1056/NEJMoa2212540

[12] Kuck KH, Lebedev DS, Mikhaylov EN, Romanov A, Gellér L, Kalējs O et al Catheter ablation or medical therapy to delay progression of atrial fibrillation: the randomized controlled atrial fibrillation progression trial (ATTEST). Europace 2021;23:362–9. 33330909 10.1093/europace/euaa298 PMC7947582

[13] Steinberg BA, Hellkamp AS, Lokhnygina Y, Patel MR, Breithardt G, Hankey GJ et al Higher risk of death and stroke in patients with persistent vs. paroxysmal atrial fibrillation: results from the ROCKET-AF trial. Eur Heart J 2015;36:288–96. 25209598 10.1093/eurheartj/ehu359 PMC4313363

[14] Qeska D, Singh SM, Qiu F, Manoragavan R, Cheung CC, Ko DT et al Variation and clinical consequences of wait-times for atrial fibrillation ablation: population level study in Ontario, Canada. Europace 2023;25:euad074. 36942997 10.1093/europace/euad074 PMC10227764

[15] Van Gelder IC, Hagens VE, Bosker HA, Kingma JH, Kamp O, Kingma T et al A comparison of rate control and rhythm control in patients with recurrent persistent atrial fibrillation. N Engl J Med 2002;347:1834–40. 12466507 10.1056/NEJMoa021375

[16] Wyse DG, Waldo AL, DiMarco JP, Domanski MJ, Rosenberg Y, Schron EB et al A comparison of rate control and rhythm control in patients with atrial fibrillation. N Engl J Med 2002;347:1825–33. 12466506 10.1056/NEJMoa021328

[17] Kirchhof P, Camm AJ, Goette A, Brandes A, Eckardt L, Elvan A et al Early rhythm-control therapy in patients with atrial fibrillation. N Engl J Med 2020;383:1305–16. 32865375 10.1056/NEJMoa2019422

[18] Ding WY, Calvert P, Gupta D, Huisman MV, Lip GYH; GLORIA-AF Investigators. Impact of early ablation of atrial fibrillation on long-term outcomes: results from phase II/III of the GLORIA-AF registry. Clin Res Cardiol 2022;111:1057–68. 35488127 10.1007/s00392-022-02022-1 PMC9424157

[19] Chun KRJ, Okumura K, Scazzuso F, Keun On Y, Kueffer FJ, Braegelmann KM et al Safety and efficacy of cryoballoon ablation for the treatment of paroxysmal and persistent AF in a real-world global setting: results from the cryo AF global registry. J Arrhythm 2021;37:356–67. 33850577 10.1002/joa3.12504 PMC8021998

[20] Földesi C, Misiková S, Ptaszyński P, Todd D, Herzet JM, Braegelmann KM et al Safety of cryoballoon ablation for the treatment of atrial fibrillation: first European results from the cryo AF Global Registry. Pacing Clin Electrophysiol 2021;44:883–94. 33813746 10.1111/pace.14237

[21] Chew DS, Black-Maier E, Loring Z, Noseworthy PA, Packer DL, Exner DV et al Diagnosis-to-ablation time and recurrence of atrial fibrillation following catheter ablation: a systematic review and meta-analysis of observational studies. Circ Arrhythm Electrophysiol 2020;13:e008128. 32191539 10.1161/CIRCEP.119.008128 PMC7359927

[22] Solimene F, Giannotti Santoro M, Stabile G, Malacrida M, De Simone A, Pandozi C et al Early rhythm-control ablation therapy to prevent atrial fibrillation recurrences: insights from the CHARISMA registry. Pacing Clin Electrophysiol 2021;44:2031–40. 34606098 10.1111/pace.14374

[23] Charitakis E, Dragioti E, Stratinaki M, Korela D, Tzeis S, Almroth H et al Predictors of recurrence after catheter ablation and electrical cardioversion of atrial fibrillation: an umbrella review of meta-analyses. Europace 2023;25:40–8. 36037026 10.1093/europace/euac143 PMC10103559

[24] Boersma L, Andrade JG, Betts T, Duytschaever M, Pürerfellner H, Santoro F et al Progress in atrial fibrillation ablation during 25 years of Europace journal. Europace 2023;25:euad244. 37622592 10.1093/europace/euad244 PMC10451004

[25] Sau A, Kapadia S, Al-Aidarous S, Howard J, Sohaib A, Sikkel MB et al Temporal trends and lesion sets for persistent atrial fibrillation ablation: a meta-analysis with trial sequential analysis and meta-regression. Circ Arrhythm Electrophysiol 2023;16:e011861. 37589197 10.1161/CIRCEP.123.011861 PMC10510845

[26] Joglar JA, Chung MK, Armbruster AL, Benjamin EJ, Chyou JY, Cronin EM et al 2023 ACC/AHA/ACCP/HRS guideline for the diagnosis and management of atrial fibrillation: a report of the American College of Cardiology/American Heart Association joint committee on clinical practice guidelines. Circulation 2024;149:e1–156. 38033089 10.1161/CIR.0000000000001193 PMC11095842

[27] Zhu W, Wu Z, Dong Y, Lip GYH, Liu C. Effectiveness of early rhythm control in improving clinical outcomes in patients with atrial fibrillation: a systematic review and meta-analysis. BMC Med 2022;20:340. 36224587 10.1186/s12916-022-02545-4 PMC9558983

[28] Chao TF, Chan YH, Chiang CE, Tuan TC, Liao JN, Chen TJ et al Early rhythm control and the risks of ischemic stroke, heart failure, mortality, and adverse events when performed early (<3 months): a nationwide cohort study of newly diagnosed patients with atrial fibrillation. Thromb Haemost 2022;122:1899–910. 35322396 10.1055/a-1807-0336

[29] Dickow J, Kany S, Roth Cardoso V, Ellinor PT, Gkoutos GV, Van Houten HK et al Outcomes of early rhythm control therapy in patients with atrial fibrillation and a high comorbidity burden in large real-world cohorts. Circ Arrhythm Electrophysiol 2023;16:e011585. 36942567 10.1161/CIRCEP.122.011585 PMC10205477

[30] Rillig A, Borof K, Breithardt G, Camm AJ, Crijns HJGM, Goette A et al Early rhythm control in patients with atrial fibrillation and high comorbidity burden. Circulation 2022;146:836–47. 35968706 10.1161/CIRCULATIONAHA.122.060274

[31] Sakamoto K, Tohyama T, Ide T, Mukai Y, Enzan N, Nagata T et al Efficacy of early catheter ablation for atrial fibrillation after admission for heart failure. JACC Clin Electrophysiol 2023;9:1948–59. 37480855 10.1016/j.jacep.2023.05.038

[32] Marrouche NF, Brachmann J, Andresen D, Siebels J, Boersma L, Jordaens L et al Catheter ablation for atrial fibrillation with heart failure. N Engl J Med 2018;378:417–27. 29385358 10.1056/NEJMoa1707855

[33] Sohns C, Fox H, Marrouche NF, Crijns HJGM, Costard-Jaeckle A, Bergau L et al Catheter ablation in end-stage heart failure with atrial fibrillation. N Engl J Med 2023;389:1380–9. 37634135 10.1056/NEJMoa2306037

[34] Kim D, Yang PS, You SC, Sung JH, Jang E, Yu HT et al Treatment timing and the effects of rhythm control strategy in patients with atrial fibrillation: nationwide cohort study. BMJ 2021;373:n991. 33975876 10.1136/bmj.n991 PMC8111568

[35] Yang E, Tang O, Metkus T, Berger RD, Spragg DD, Calkins HG et al The role of timing in treatment of atrial fibrillation: an AFFIRM substudy. Heart Rhythm 2021;18:674–81. 33383228 10.1016/j.hrthm.2020.12.025

[36] Lawin D, Kuhn S, Schulze Lammers S, Lawrenz T, Stellbrink C. Use of digital health applications for the detection of atrial fibrillation. Herzschrittmacherther Elektrophysiol 2022;33:373–9. 35960358 10.1007/s00399-022-00888-2

