# Supplementary material for: Impact of atrial fibrillation diagnosis-to-ablation time on 24-month efficacy and safety outcomes in the Cryo Global Registry
Source: Europace. 2025 Jan 21;27(2):euaf008. doi: 10.1093/europace/euaf008 (PMC11795645; doi:10.1093/europace/euaf008)
Supplement: euaf008_Supplementary_Data [file euaf008_supplementary_data.docx]

**Supplement**

**Manuscript:** Impact of Atrial Fibrillation Diagnosis-to-Ablation Time on 24-Month Efficacy and Safety Outcomes in the Cryo Global Registry. Lawin *et al*.

**Figure S1: Patient disposition**


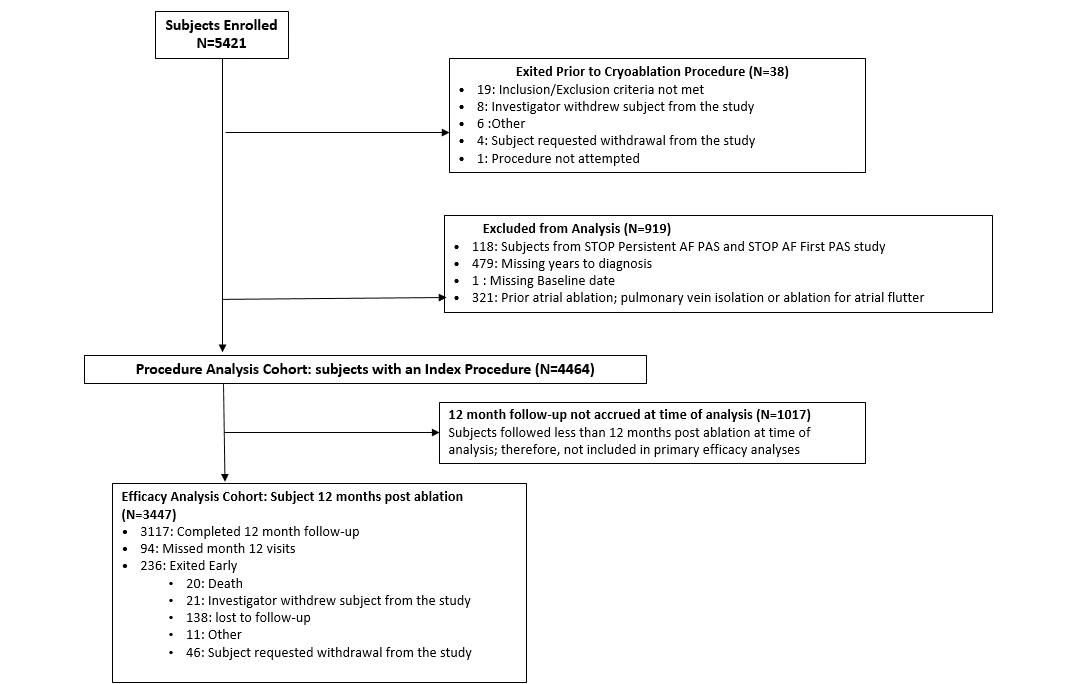


**Center experience**

**Table S1** displays the distribution of patients enrolled in the early ablation group versus late ablation group. Centers with more experience enrolled a higher percentage of early ablation patients (<0.01). Centers with more prior cryoablation experience did not impact efficacy (**Table S2**, p=0.08) or safety (**Table S3**, p = 0.52). Efficacy was consistently higher in the early ablation group, and safety rates were consistently lower in the early ablation group, regardless of center experience.

**Table S1. Prior Cryoballoon Experience**

|  |  |  | **Number (%) enrolled patients** | |  |
| --- | --- | --- | --- | --- | --- |
| **Number of Cryo Procedures in Prior Year^1^** | **Number of Centers^2^** | **Total patients** | **Ablation ≤ 12 months from diagnosis** | **Ablation >12 months from diagnosis** | **p-value** |
| **≤ 25** | 29 | 674 | 283 (42.0%) | 391 (58.0%) |  |
| 26 - 50 | 23 | 559 | 223 (39.9%) | 336 (60.1%) |  |
| 51 - 100 | 28 | 748 | 295 (39.4%) | 453 (60.6%) | <0.01^3^ |
| 101 - 200 | 20 | 713 | 364 (51.1%) | 349 (48.9%) |  |
| > 200 | 16 | 650 | 346 (53.2%) | 304 (46.8%) |  |
| ^1^ Number of cryoballoon procedures completed at study center in the year prior to site activating for the study.  ^2^ 116 / 121 centers with data available  ^3^Cochran-Armitage Test | | | | | |

**Table S2. Center Experience - Efficacy**

|  |  | **N patients** | | **12-Month Efficacy** | |  |
| --- | --- | --- | --- | --- | --- | --- |
| **Number of Cryo Procedures in Prior Year^1^** | **Number of Centers^2^** | **Ablation ≤ 12 months from diagnosis** | **Ablation >12 months from diagnosis** | **Ablation ≤ 12 months from diagnosis** | **Ablation >12 months from diagnosis** | **p-value** |
| **≤ 25** | 29 | 283 | 391 | 85.8% (95% CI: 81.0 – 89.5%) | 75.2% (95% CI: 70.5 – 79.2%) |  |
| **26 - 50** | 23 | 223 | 336 | 85.8% (95% CI: 80.3 – 89.8%) | 83.7% (95% CI: 79.3 – 87.3%) |  |
| **51 - 100** | 28 | 295 | 453 | 84.4% (95% CI: 79.6 – 88.1%) | 77.9% (95% CI: 73.7 – 81.5%) | 0.08^3^ |
| **101 - 200** | 20 | 364 | 349 | 87.9% (95% CI: 83.8 – 91.0%) | 82.0% (95% CI: 77.4 – 85.8%) |  |
| **> 200** | 16 | 346 | 304 | 85.2% (95% CI: 80.7 – 88.8%) | 85.9% (95% CI: 81.1 – 89.6%) |  |
| ^1^ Number of cryoballoon procedures completed at study center in the year prior to site activating for the study.  ^2^ 116 / 121 centers with data available  ^3^ Interaction test, assessing the effect of center experience on early versus late ablation efficacy. Tested with a Cox regression model, with ablation timing subgroup, center experience, and ablation timing subgroup*center experience interaction term as covariates in the model. | | | | | | |

**Table S3. Center Experience - Safety**

|  |  | **N patients** | | **N (%) patients with safety event** | |  |
| --- | --- | --- | --- | --- | --- | --- |
| **Number of Cryo Procedures in Prior Year^1^** | **Number of Centers^2^** | **Ablation ≤ 12 months from diagnosis** | **Ablation >12 months from diagnosis** | **Ablation ≤ 12 months from diagnosis** | **Ablation >12 months from diagnosis** | **p-value** |
| **≤ 25** | 29 | 283 | 391 | 5 (1.8%) | 11 (2.8%) |  |
| **26 - 50** | 23 | 223 | 336 | 4 (1.8%) | 8 (2.4%) |  |
| **51 - 100** | 28 | 295 | 453 | 13 (4.4%) | 24 (5.3%) | 0.52^1^ |
| **101 - 200** | 20 | 364 | 349 | 11 (3.0%) | 9 (2.6%) |  |
| **> 200** | 16 | 346 | 304 | 4 (1.2%) | 12 (3.9%) |  |
| ^1^ Number of cryoballoon procedures completed at study center in the year prior to site activating for the study.  ^2^ 116 / 121 centers with data available  ^3^ Interaction test, assessing the effect of center experience on early versus late ablation safety rates. Tested with a logistic regression model, with ablation timing subgroup, center experience, and ablation timing subgroup*center experience interaction term as covariates in the model. | | | | | | |

Analysis methods:

The association between prior cryoablation experience and number of early ablation patients (ablation ≤ 12 months from diagnosis) enrolled was assessed with a Cochran-Armitage trend test (Table 1). Cox regression was utilized to test for center experience effect on efficacy. AF/AFL/AT recurrence was the dependent variable in the model and early/later ablation subgroup, number of prior cryoablations at the study center, and early/later ablation subgroup indicator*number of prior cryoablations interaction term were included as covariates. Center experience was tested with the Wald chi-square test statistic for the interaction term (Table S2). Logistic regression was utilized to test for center experience effect on safety event rates. Safety event was the dependent variable in the model and early/later ablation subgroup, number of prior cryoablations at the study center, and early/later ablation subgroup indicator*number of prior cryoablations interaction term were included as covariates. Center experience was tested with the Wald chi-square test statistic for the interaction term (Table S3).
